# Multimedia Cryptosystem for IoT Applications Based on a Novel Chaotic System around a Predefined Manifold

**DOI:** 10.3390/s22010334

**Published:** 2022-01-03

**Authors:** Li Li, Ahmed A. Abd El-Latif, Sajad Jafari, Karthikeyan Rajagopal, Fahimeh Nazarimehr, Bassem Abd-El-Atty

**Affiliations:** 1 Shenzhen Institute of Information Technology, Shenzhen 518172, China; lili_sziit2014@163.com; 2 Department of Mathematics and Computer Science, Faculty of Science, Menoufia University, Shebin El-Koom 32511, Egypt; 3 Department of Biomedical Engineering, Amirkabir University of Technology, 424 Hafez Ave., Tehran 15875-4413, Iran; sajadjafari83@gmail.com (S.J.); fahimenazarimehr@yahoo.com (F.N.); 4 Health Technology Research Institute, Amirkabir University of Technology, No. 350, Hafez Ave., Valiasr Square, Tehran 159163-4311, Iran; 5 Center for Nonlinear Systems, Chennai Institute of Technology, Chennai 600069, India; karthikeyan.rajagopal@citchennai.net; 6 Department of Computer Science, Faculty of Computers and Information, Luxor University, Luxor 85957, Egypt; bassem.abdelatty@fci.luxor.edu.eg

**Keywords:** multimedia cryptosystem, IoT applications, chaotic systems, image security

## Abstract

Multimedia data play an important role in our daily lives. The evolution of internet technologies means that multimedia data can easily participate amongst various users for specific purposes, in which multimedia data confidentiality and integrity have serious security issues. Chaos models play an important role in designing robust multimedia data cryptosystems. In this paper, a novel chaotic oscillator is presented. The oscillator has a particular property in which the chaotic dynamics are around pre-located manifolds. Various dynamics of the oscillator are studied. After analyzing the complex dynamics of the oscillator, it is applied to designing a new image cryptosystem, in which the results of the presented cryptosystem are tested from various viewpoints such as randomness, time encryption, correlation, plain image sensitivity, key-space, key sensitivity, histogram, entropy, resistance to classical types of attacks, and data loss analyses. The goal of the paper is proposing an applicable encryption method based on a novel chaotic oscillator with an attractor around a pre-located manifold. All the investigations confirm the reliability of using the presented cryptosystem for various IoT applications from image capture to use it.

## 1. Introduction

Developments in the Internet of Things (IoT), cloud computing, and fifth-generation network technologies make multimedia data easy to share with various users for specific purposes. In this context, the sharing of multimedia data suffers from serious security issues [1,2,3,4]. Multimedia data can be secured via executing one of the protection techniques: information hiding and data encryption mechanisms. Encryption techniques aim to transform multimedia data from an understood pattern to an incomprehensible form. Recently, chaos systems have played an important role in designing robust multimedia data encryption mechanisms and secure communication.

Chaotic oscillations are very complex dynamics, and there are many ambiguities about them. Many studies try to clarify the creation of chaotic dynamics [5]. Formerly, there was an idea that chaotic dynamics are related to saddle equilibria [6]. Then, some chaotic systems were proposed that do not have a saddle point [7]. Recently, many studies have been done on the investigation of chaotic oscillators with different properties [8]. Some examples of chaotic systems with various types of equilibria are given [5]. A chaotic oscillator with a line of equilibria was studied in [9]. In [10], hyperchaotic dynamics in a system without equilibria were discussed. The application of a chaotic oscillator with no equilibria was studied in [11]. Multistability is an exciting property of a dynamic system. The multistability of a new version of the Chua system was discussed in [12]. Furthermore, multistability as a feature of a hyperjerk was studied in [13]. Extreme multistability is a feature in which the system has a complete bifurcation diagram by changing initial conditions, not parameters [14]. Memristive neural models were studied in [15]. Chaotic flows have various engineering applications such as chaotic circuit [16].

Proposing chaotic systems with dynamics around a predefined manifold has been an exciting topic. In this paper, a novel chaotic oscillator is presented. The oscillator has a particular property in which the chaotic dynamics are around pre-located manifolds. Various dynamics of the oscillator are studied. The chaotic attractor and the predefined manifolds are discussed, and their relation with equilibrium points is investigated. Studying the bifurcation diagrams of the proposed system by different initiation methods shows the multistability of the system in some intervals of the bifurcation parameter. Lyapunov exponents of the oscillator are studied to show the interval of chaotic dynamics. In the multistability region, the exciting basin of attraction of various attractors is studied.

Digital images are widely used for representing multimedia data in numerous applications. The complexity of chaotic time series makes them a proper choice for image encryption and secure communication [17,18,19]. Nonlinear methods are useful in image encryption [20,21,22]. The application of a chaotic oscillator in fingerprint encryption was discussed in [23]. In [24], image encryption using a chaotic map was studied. Moreover, the encryption of medical images using a chaotic map was discussed in [25], and applying an exponential chaotic oscillator in secure communication was investigated in [26].

Based on the nonlinear features of the presented chaotic oscillator system, we present a novel image cryptosystem for IoT applications, in which the results of the presented cryptosystem are tested from various viewpoints. All the investigations show the reliability of the image cryptosystem for various IoT applications.

The presented contributions of this work can be outlined as follows:

- Presenting a novel chaotic oscillator, in which the chaotic dynamics are around pre-located manifolds.
- Designing a novel image cryptosystem for IoT applications, of which the design is based on the nonlinear features of the presented chaotic oscillator system.

## 2. Proposed Framework for IoT Environment

**Figure 1:**
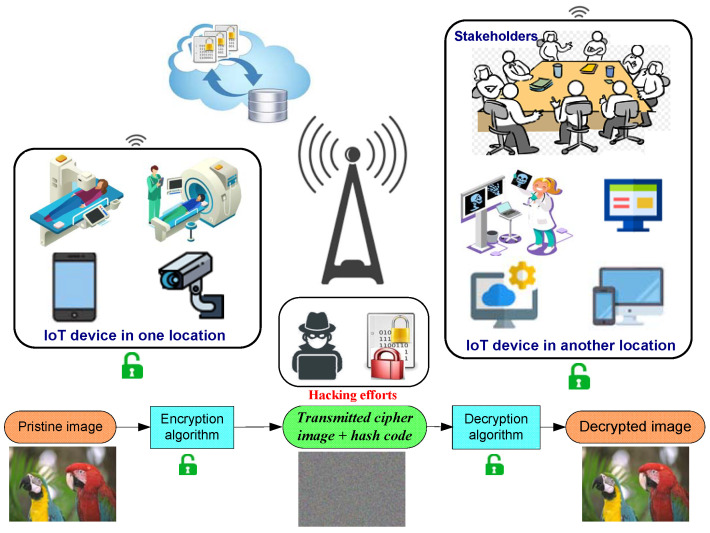
Proposed framework for the secure transmission of multimedia data in IoT environment.

Multimedia data, such as images, audio, and video are growing rapidly as an essential avenue for the representing, sharing, and storage of data in our daily lives. The evolution of internet technologies makes multimedia data can easily stored in cloud storage and shared amongst various users for specific goals. In this context, the confidentiality and integrity of multimedia data suffer from serious security issues. Therefore, we proposed a new framework for IoT applications to store and share digital images, in which image data confidentiality and integrity are achieved. The proposed framework is presented in Figure 1.

The presented image cryptosystem can be utilized in different application fields, such as the medical sector, surveillance systems, personal data protection, etc. In the medical sector, the presented image cryptosystem can be utilized for the secure transmission of medical images from their origin to the intended stakeholders for analyzing, assessing, and treat patients. In surveillance systems, when the system detects any movement in the camera location, the video frames are captured and encrypted via the presented cryptosystem then sent the encrypted data to the intended stakeholders for analyzing and taking the appropriate decision. Moreover, the presented cryptosystem can be utilized for storing multimedia data to cloud storage then sharing/downloading it up to require.

To maintain the integrity of the transmitted data via our presented cryptosystem, we utilized a hashing algorithm like SHA-256. The hashing algorithm is employed to get the hash code for the appended secret key with the cipher multimedia (secret key + cipher data), for making SHA-256 a keyed hash algorithm, then the generated hash code is sent with the cipher data. Upon the intended user downloaded the encrypted data and receiving the hash code, the hash value is computed for the received cipher data with the secret key, and investigate if the generated hash value is the same as the received hash code or not. If the two hash values are the same, then there are no changes in the transmitted cipher data, and the integrity of transmitted data has been achieved.

## 3. Proposed Chaotic Oscillator System

In this paper, a chaotic oscillator with a unique feature is proposed as Equation (1).

(1) $$\begin{array}{c} \dot{x}=z\\ \dot{y}=x^{2}+y^{2}-1\\ \dot{z}=-2x-y+az+xy \end{array}$$

**Figure 2:**
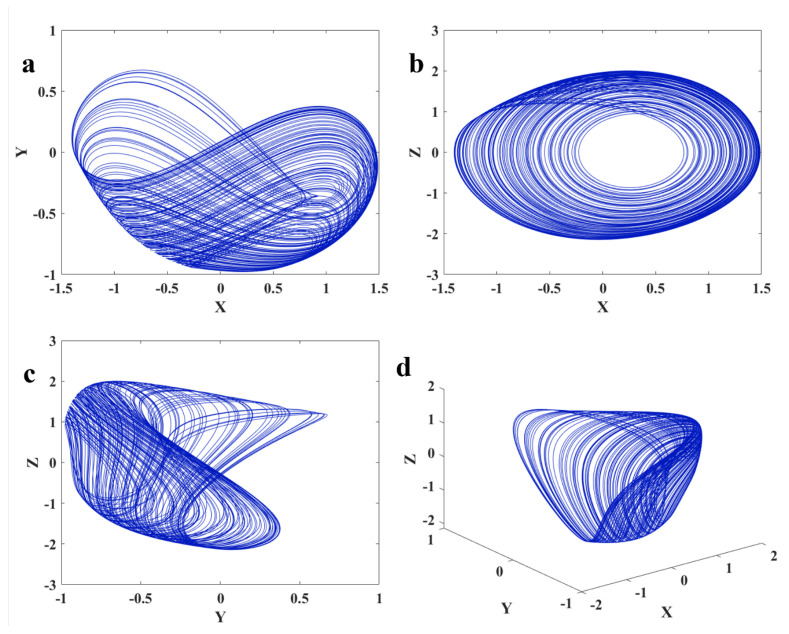
The chaotic dynamics of Equation (1) with a=0 and $\left(x_{0},y_{0},z_{0}\right)=\left(0,0,0\right)$ in (**a**) X−Y; (**b**) X−Z; (**c**) Y−Z; (**d**) X−Y−Z.

**Figure 3:**
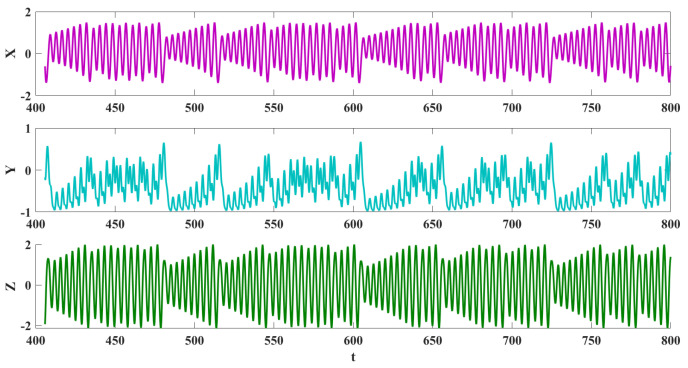
Time series of Equation (1) with a=0 and $\left(x_{0},y_{0},z_{0}\right)=\left(0,0,0\right)$.

**Figure 4:**
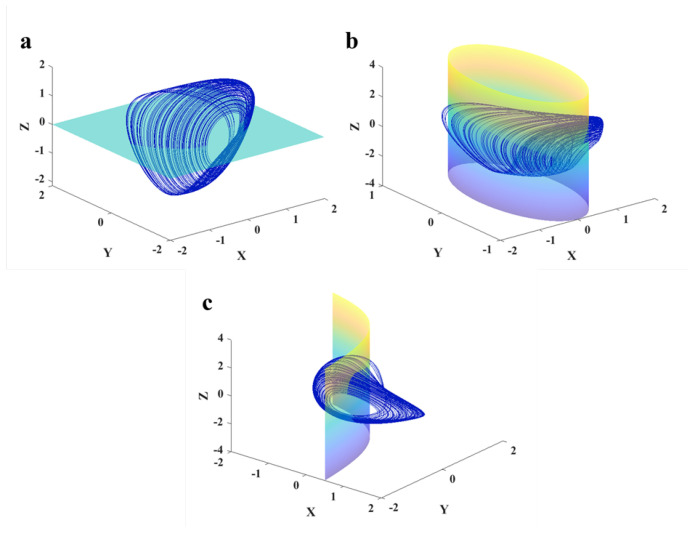
3D chaotic dynamics of Equation (1) and the three pre-located manifolds (**a**–**c**).

The system presents a chaotic dynamic in a=0, and initial values $\left(x_{0},y_{0},z_{0}\right)=\left(0,0,0\right)$. Its Lyapunov exponents are $\left(0.1322,0,-0.9518\right)$. Three projections and the 3D chaotic solution of the oscillator are shown in Figure 2. Figure 3 presents signals of various variables for the chaotic dynamic. In order to have bounded solutions, the derivative of variables should be zeros. So, the system’s dynamics should be around the pre-located manifolds as $z=0,x^{2}+y^{2}=1,$ and $y=\frac{2x}{x-1}$. The chaotic dynamic and these manifolds are plotted in Figure 4.

## 4. Dynamical Properties of the Oscillator

### 4.1. Equilibrium Points

To calculate the equilibrium points of the oscillator, all of its derivatives should be zeros simultaneously. So we can tell that the intersections of the three studied manifolds are the equilibria of the system. Equation (1) has two equilibrium points as $E_{1}=\left(0.3213,-0.947,0\right),E_{2}=\left(-0.6323,0.7747,0\right)$. In parameter a=0, the eigenvalues of the oscillator for $E_{1}$ are $\left(-1.9582,0.0322\pm 1.7528i\right)$, and for $Eq_{2}$ are $\left(1.9576,-0.2041\pm 1.4081i\right)$. So, the equilibrium points are saddle points. It means that the chaotic dynamics are self-excited.

### 4.2. Bifurcation Diagram and Lyapunov Exponents

**Figure 5:**
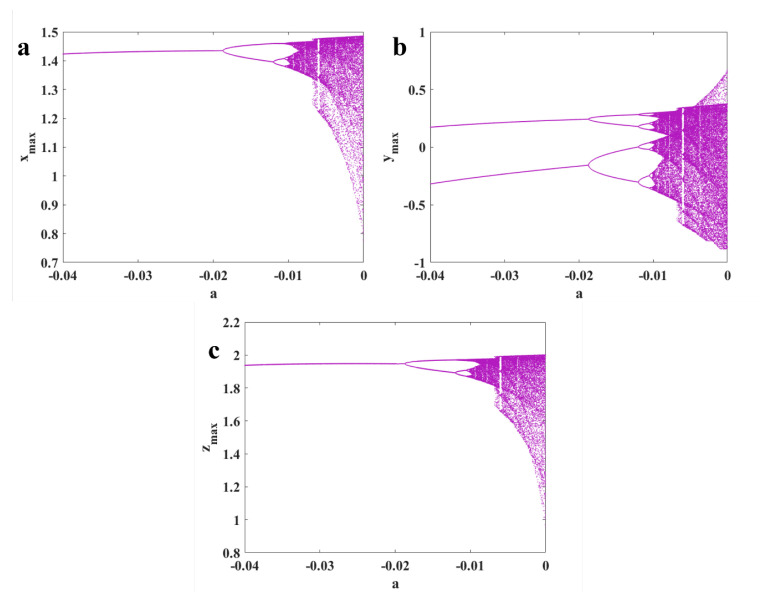
Bifurcation diagram of Equation (1) by changing parameter *a* and backward continuation method; maximum values of (**a**) *x* variable; (**b**) *y* variable; (**c**) *z* variable.

**Figure 6:**
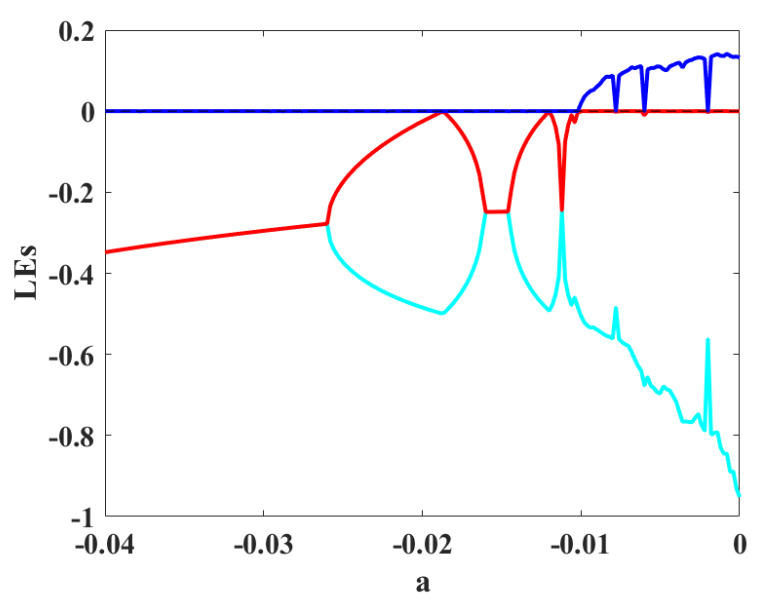
The diagram of LEs by changing parameter *a*.

To study different dynamics of the oscillator, the bifurcation diagram is presented in Figure 5. In these diagrams, the maximum values of the three variables are plotted by changing bifurcation parameter *a*. The diagram is plotted by the backward continuation method, and the first set of initial conditions are $\left(0,0,0\right)$. A period-doubling route to chaos can be seen in the bifurcation diagram. To confirm the existence of chaos, Lyapunov exponents (LEs) are computed with run time 20,000. Figure 6 presents the diagram of LEs by changing parameter *a*, corresponding to the bifurcation diagram. One positive LE shows chaotic behaviors.

### 4.3. Multistability Analysis

**Figure 7:**
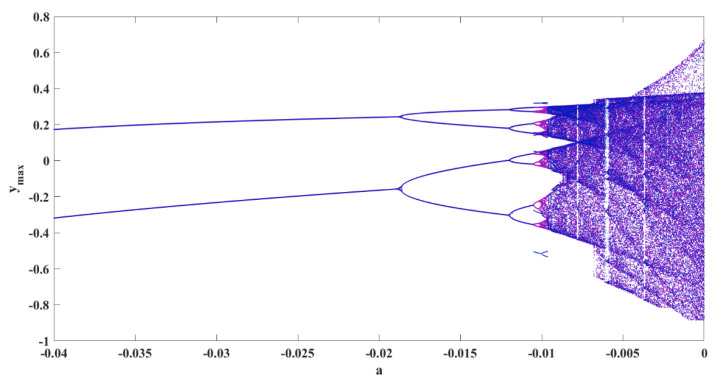
Bifurcation diagram of the oscillator by changing parameter *a*; bifurcation with backward continuation method is shown in purple and with constant initial conditions is shown in blue.

Multistability is one of the exciting features of dynamical systems. It means that two different sets of initial conditions result in two different attractors. The multistability of the oscillator is examined by plotting bifurcation diagrams using two different initiation methods, backward and constant initiation. Figure 7 shows the backward bifurcation in purple and bifurcation with constant initial conditions in blue. The results reveal the coexisting attractors in the interval $a\in \left[-0.0105,-0.00975\right]$, since the two diagrams with different initiation methods are not the same.

### 4.4. Basin of Attraction

**Figure 8:**
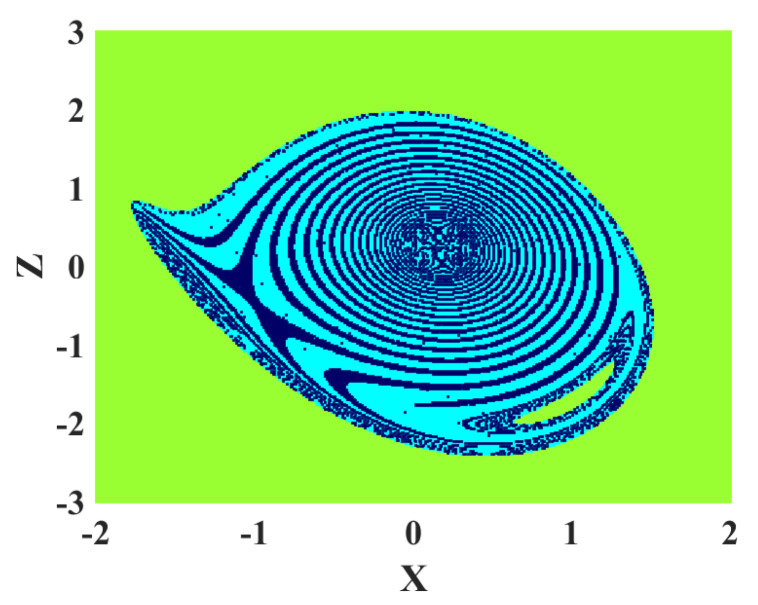
Basin of attraction of the oscillator; green, dark blue, and cyan regions show the basin of attraction for unbounded, periodic, and chaotic solutions, respectively.

After revealing the coexisting attractors, investigating the basin of attraction of each attractor is interesting. Figure 8 shows the basin of attraction of the oscillator in parameter a=−0.0099. It can be seen in Figure 7 that two periodic and chaotic dynamics coexist in this parameter. In Figure 8, the green, dark blue, and cyan regions show the basin of attraction for unbounded, periodic, and chaotic solutions.The basin of attractions of different attractors are entangled with each other.

## 5. The Proposed Image Encryption Approach

The protection of data represented by images can be achieved via image data protection techniques like image encryption, image data hiding, or mixing between them [27,28]. Spring from the presented chaotic system’s benefits, we propose a new image encryption approach, which necessitates an adaptation for our chaotic map as presented in Equation (2).

(2) $$\left\{\begin{array}{c} x_{i+1}=z_{i}mod1\\ y_{i+1}=\left(x_{i}^{2}+y_{i}^{2}-1\right)mod1\\ z_{i+1}=\left(-2x_{i}-y_{i}+az_{i}+x_{i}y_{i}\right)mod1 \end{array}\right.$$

**Figure 9:**
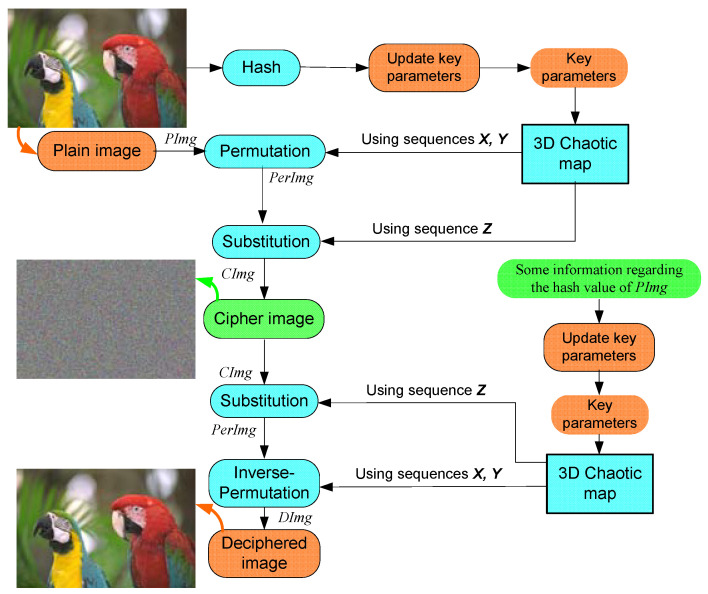
Description of the proposed cryptosystem for multimedia images.

The presented image cryptosystem employs the benefits of our chaotic system to generate three pseudo-random sequences. The first two sequences are used to permute the plain image. Then the last sequence is utilized to substitute the permuted image for constructing the cipher image. The multimedia image cryptosystem is described in Figure 9, and the encryption procedure is listed in the following steps.

(1) Perform the hash function SHA256 on the plain image (*PImg*) to get the hash value (V).
(2) Convert *V* into 32 integer numbers (*$v_{1}$, $v_{2}$, $v_{3}$, ..., $v_{32}$*) each of 8-bit, then obtain 3 decimal numbers from these integers as follows.
  $$\begin{array}{c} D_{1}=\frac{v_{1}⊕v_{2}⊕\cdots ⊕v_{11}}{256}\\ D_{2}=\frac{v_{12}⊕v_{10}⊕\cdots ⊕v_{22}}{256}\\ D_{3}=\frac{v_{23}⊕v_{18}⊕\cdots ⊕v_{32}}{256} \end{array}$$
(3) Choose initial values for key parameters ( $x_{initial}$, $y_{initial}$, $z_{initial}$) and update these keys using $D_{1}$, $D_{2}$, and $D_{3}$.
  $$\begin{array}{c} x_{0}=\frac{x_{\mathrm{initial}}+D_{1}}{2}\\ y_{0}=\frac{y_{\mathrm{initial}}+D_{2}}{2}\\ z_{0}=\frac{z_{\mathrm{initial}}+D_{3}}{2} \end{array}$$
(4) Iterate the chaotic system (2) for H×W×L times using the updated key parameters ($x_{0}$, $y_{0}$, $z_{0}$, *a*) for generating 3 sequences (*X*, *Y*, *Z*), in which H×W×L is the size of *PImg*.
(5) Add the values of *X* to the values of *Y* as sequence *W*, then sort the values of *W* from the smallest to the largest as sequence *S*, and obtain the index *S* in *W* as *PrVc*.
(6) Reshape the plain image (*PImg*) into a vector (*PImgVc*) and permute *PImgVc* using the produced vector *PrVc* as follows.
  PerImgVc(i)=PImgVc(PrVc(i))
  fori=1,2,...,H×W×L
(7) Construct the key sequence (*K*) by adapting the sequence *Z* into integers.
  $$K=fix(Z\times 10^{12})\;\;\mathrm{mod}\;\;\;256$$
(8) Perform Bitwise-Xor operation on the permuted vector *PerImgVc* and *K* to construct the cipher image *CImg*.
  CImgVc=PerImgVc⊕K
  CImg=reshape(CImgVc,H,W,L)

## 6. Experimental Outcomes

**Figure 10:**
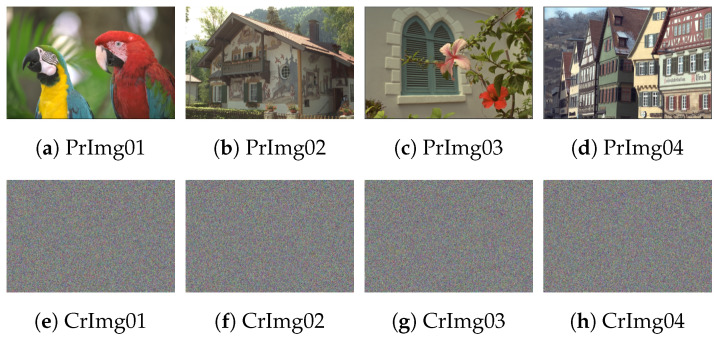
Experimented image dataset of dimensional 768 × 512, in which the first row denotes the plain images, while the second row describes the corresponding ciphered images.

The utilized dataset of images is obtained from the Kodak database [29]. It consists of four images named Macaws, Chalet, Window, and Houses, with size 768 × 512 (see Figure 10). The utilized initial values to iterate the 3D chaotic map are $x_{initial}$ = 0.6275, $y_{initial}$ = 0.3854, $z_{initial}$ = 0.7261, a= 0.

The effectiveness of any image cryptosystem depends essentially on performance (how fast we can encrypt an image on a defined computer) and resistance to various attacks: such as brute force, linear and differential cryptanalysis, statistical cryptanalysis, etc.). These two essential properties are discussed in the following subsections to show the effectiveness of the presented image cryptosystem.

### 6.1. Time Efficiency

To demonstrate the effectiveness of the presented cryptosystem in terms of time encryption, Table 1 stated a superficial comparison of time encryption for the presented cryptosystem with other related encryption algorithms, as reported in [30,31,32]. From the stated information in Table 1, we can deduce that our cryptosystem is more superb than other ones in terms of time encryption.

### 6.2. Randomness Analysis

For testing the randomness of the created sequence constructed the cipher image, we perform NIST SP 800-22 tests composed of fifteen tests. These tests are applied on a 2,000,000-bit of the constructed Cipher–Macaws image and its utilized key-stream. The outcomes are provided in Table 2, which declares that all NIST SP 800-22 tests are passed successfully. Consequently, the presented 3D chaotic system can be reliable in designing various modern cryptographic applications.

### 6.3. Correlation Analysis

**Figure 11:**
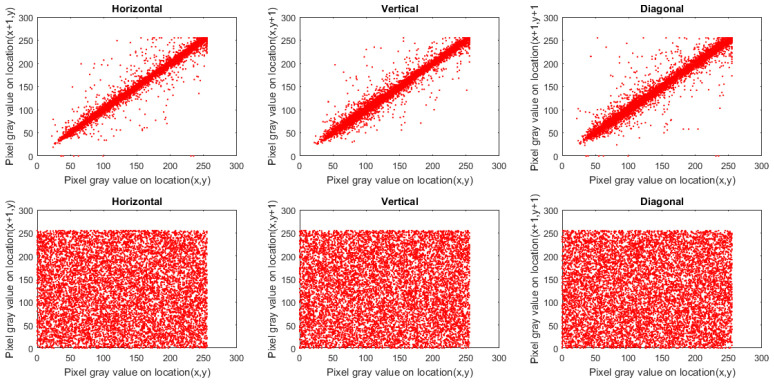
Plots of correlation distribution (in each direction) for Macaws image (Red channel), in which the first row denotes the plain Macaws image, while the bottom row signifies the ciphered Macaws image.

**Figure 12:**
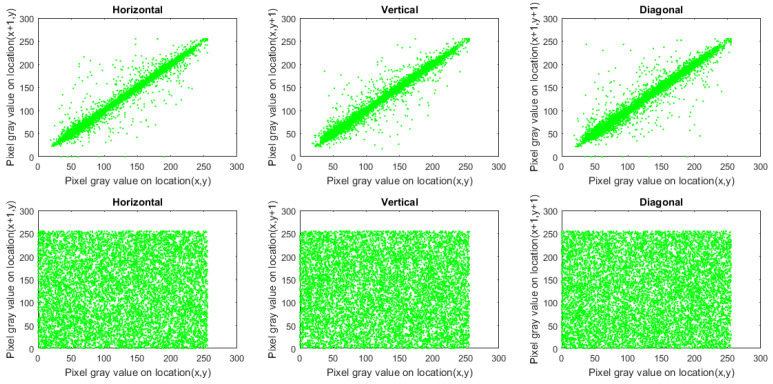
Plots of correlation distribution (in each direction) for Macaws image (Green channel), in which the first row denotes the plain Macaws image, while the bottom row signifies the ciphered Macaws image.

**Figure 13:**
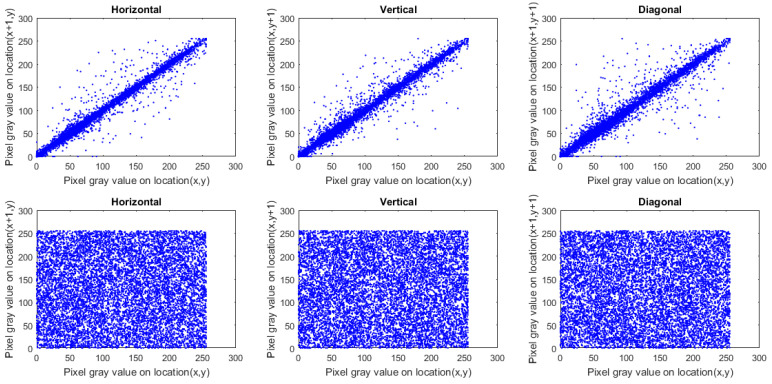
Plots of correlation distribution (in each direction) for Macaws image (Blue channel), in which the first row denotes the plain Macaws image, while the bottom row signifies the ciphered Macaws image.

To study the perception of an image, we employ the correlation coefficient (CC) of adjacent pixels. Ordinary images possess CC near 1 in each direction. Cipher images (constructed using a robust-designed image cryptosystem) should be approximately 0. To calculate CC for the original and encrypted images, we picked 10,000 pairs of adjacent pixels at random in every direction.

(3) $$CC=\frac{\sum_{x=1}^{A}\left(p_{x}-\bar{p}\right)\left(c_{x}-\bar{c}\right)}{\sqrt{\sum_{x=1}^{A}{\left(p_{x}-\bar{p}\right)}^{2}\sum_{x=1}^{N}{\left(c_{x}-\bar{c}\right)}^{2}}}$$

where *A* indicates the entire number of adjacent pixel pairs and $c_{x}$, $p_{x}$ indicate the adjacent pixels. Table 3 presents the outcomes of CC for encrypted images and plain ones, in which the CC for cipher images is approximately 0. Additionally, Figure 11, Figure 12 and Figure 13 plot the distribution of correlation per direction in Macaws image and its encrypted one. From the outcomes provided in Table 3 and shown in Figure 11, Figure 12 and Figure 13, no valuable information was gained regarding the plain image by analyzing CC values.

### 6.4. Differential Analysis

Plain-image sensitivity refers to any tiny modifications on the plain image, resulting in a massive difference for the cipher image. To test plain-image sensitivity for our image cryptosystem, we employ NPCR (“Number of Pixel Change Rate”) and UNCI (“Unified Average Changing Intensity”), which are represented as follows,

(4) $$\begin{array}{c} NPCR=\frac{\sum_{i;j}Df\left(i,j\right)}{A}\times 100\%,\\ Df\left(i,j\right)=\left\{\begin{array}{c} 0\;\;if\;\;C1(i,j)=C2(i,j)\\ 1\;\;if\;\;C1(i,j)\neq C2(i,j) \end{array}\right. \end{array}$$

(5) $$UACI=\frac{1}{A}\left(\sum_{i,j}\frac{\left|C1(i,j)-C2(i,j)\right|}{255}\right)\times 100\%$$

where *A* denotes the full number of image pixels and C1, C2 denote the two ciphered images for a plain image differs in one bit. The outcomes of NPCR and UNCI are stated in Table 4. It demonstrates our image cryptosystem enjoys high sensitivity to slight modifications in the original image.

### 6.5. Key-Space Analysis

Multifarious secret keys that can be utilized in brute force attacks are known as key-space. By the benchmark stated in [33], the key-space ought to be larger than $2^{100}$ to demonstrate sufficient security against brute-force attacks. The presented image cryptosystem uses initial key parameters ($x_{initial}$, $y_{initial}$, $z_{initial}$, and *a*) to generate the chaotic sequences utilized in encryption and decryption procedures. By assuming the precision of computation for digital devices is $10^{-16}$, then the key-space for the presented mechanism is ≃$2^{213}$, which is immense sufficiently for any cryptographic algorithm.

### 6.6. Key Sensitivity Analysis

**Figure 14:**
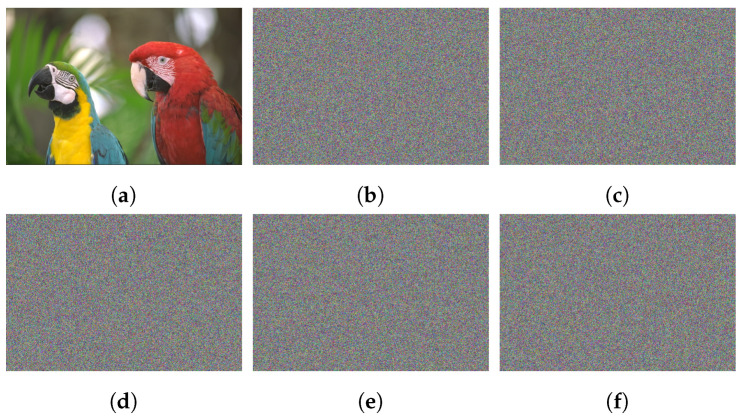
Outcomes of decrypting Cipher–Macaws image using slight changes in the confidential key. (**a**) The confidential key; (**b**) The confidential key except $x_{initial}$ = 0.627500000000001; (**c**) The confidential key except $y_{initial}$ = 0.3854000000000001; (**d**) The confidential key except $z_{initial}$ = 0.7261000000000001; (**e**) The confidential key except $x_{initial}$ = 0.627499999999999; (**f**) The confidential key except $y_{initial}$ = 0.38539999999999.

It is defined as the sensitivity of the decryption to the secret key. It is a necessary measure to guarantee the reliability of any cryptosystem. For evaluating the suggested image cryptosystem’s key sensitivity, the Cipher-Macaws image is deciphered many times using slight changes in the secret key as displayed in Figure 14.

### 6.7. Histogram Analysis

**Figure 15:**
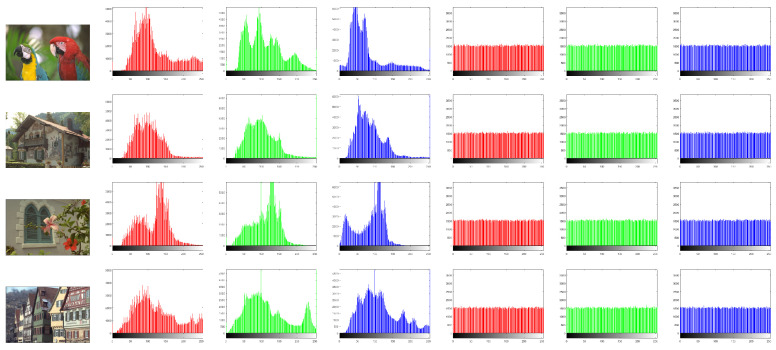
Plots of histograms for experimented images, in which the left three columns except the first one represent the histograms of plain images, while the right three columns represent the histograms of cipher images.

To evaluate the pixel values’ distribution in the encrypted images, the histogram test is employed. A proper image cryptosystem has to guarantee the identical distribution for varied cipher images. Figure 15 plots the histograms of the studied images. The histograms of the plain images are not similar, while the histograms of their related ciphered ones are uniform. Additionally, we applied chi-square ($\chi^{2}$) analysis to guarantee the histogram results.

(6) $$\chi^{2}=\sum_{j=0}^{255}\frac{{\left(f_{j}-D\right)}^{2}}{D}$$

where *D* denotes the image dimension and $f_{j}$ represents the frequency of the pixel value *j*. By considering the significant level β = 0.05, then $\chi_{\beta }^{2}\left(255\right)=293.3$ is obtained. For an image, when the $\chi^{2}$ value is less than $\chi_{\beta }^{2}\left(255\right)$, then the histogram of this image is uniform. Table 5 provides the outcomes of $\chi^{2}$ for the studied images, in which the $\chi^{2}$ outcomes for all encrypted images are less than $\chi_{\beta }^{2}\left(255\right)$. So, the proposed image cryptosystem can withstand attacks of histogram analysis.

### 6.8. Entropy Analysis

To evaluate the bit distribution for each level of the pixel values of the encrypted image, the global entropy is employed as follows:

(7) $$E\left(X\right)=-\sum_{j=0}^{255}r\left(x_{j}\right)\log_{2}\left(r(x_{j})\right)$$

where $r(x_{j})$ is the probability of $x_{j}$. The possible values of a grayscale image are $2^{8}$, so the optimal entropy is 8-bit. Subsequently, to assess the efficacy of the suggested cryptosystem, the entropy of the ciphered images should be near to 8. The global entropy neglects the assessment of real randomness for cipher images. Therefore, we employ local entropy to assess the actual randomness for cipher images which can be computed via the average global entropies for no overlapping blocks (the size of each block is 44 × 44). Table 6 shows the results of local and global entropies for the experimented images, in which all outcomes of information entropy for cipher images are approximately 8-bit. Consequently, the proposed cryptosystem is robust against entropy attacks.

### 6.9. Classical Types of Attack

**Figure 16:**
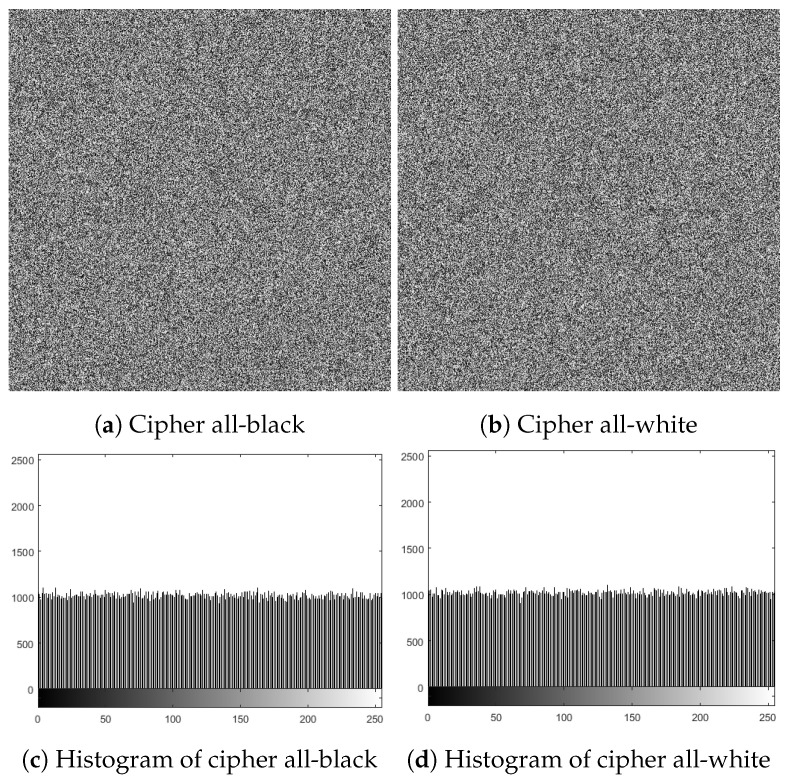
Ciphers of all-black and all-white images, and their analog histograms.

In general, the cryptanalysis of a cryptosystem assumes that cryptanalysts fully understand the structure of the cryptosystem and know all things about the encryption and decryption algorithms except the secret key utilized in encryption and decryption procedures. There are four kinds of attacks: ciphertext-only, chosen-ciphertext, chosen-plaintext, and known-plaintext [30]. The chosen-plaintext attack is considered to be the most effective attack in which the cyberpunk has temporary access to the cryptosystem and can create the ciphertext associated with the selected plaintext. If an encryption algorithm is able to resist the chosen-plaintext attack, it has the capability to resist other kinds of attacks. In the proposed encryption algorithm, if any of the initial keys ($x_{initial}$, $y_{initial}$, $z_{initial}$, and *a*) have a slight modification, the outcome will change vastly. Furthermore, our encryption approach applies SHA256 to the plain image for updating the initial parameters, so that our cryptosystem not only depends on the secret key but also on the plain image. The cryptanalyst attempts to acquire useful information about the secret key utilizing all-white and all-black images, as they can disable the task of permutation/substitution procedures. The affiliated cipher images for the all-white and all-black images and their related histograms are provided in Figure 16, in which no visual information can be acquired from these cipher images, while Table 7 supplies some statistical analyses for these images. As a result, our cryptosystem has the capability to resist linear cryptanalysis.

### 6.10. Occlusion Analysis

**Figure 17:**
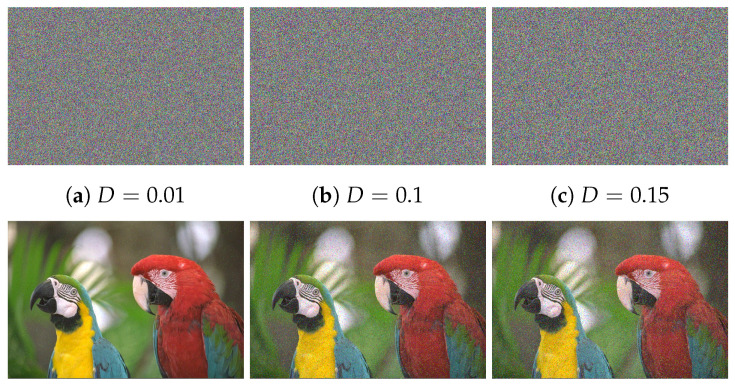
Outcomes of noise attack, in which the top row refers to the defective Cipher–Macaws image by adding Salt and Pepper noise with variable densities (D) while the bottom row represents the corresponding decipher image.

**Figure 18:**
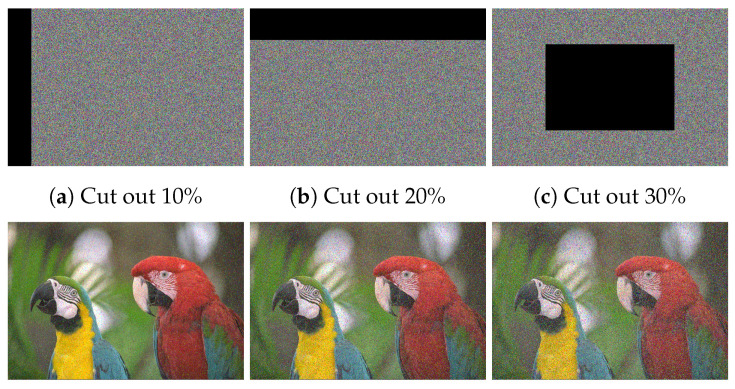
Outcomes of data loss attack, in which the top row refers to the defective Cipher–Macaws image by performing a cutting block for data with various sizes, while the bottom row represents the corresponding decipher image.

It is significant that most of the data transmission networks are noisy channels. Once data are transmitted over noisy networks, it is probably distorted by noise or data loss attacks. So, a well-designed cryptosystem should withstand data loss and noise attacks. For investigating the proposed image cryptosystem for facing data loss and noise attacks, we defect the Cipher–Macaws image via performing a cutting block for data with various sizes or applying Salt and Pepper noise with variable densities. Then we decipher the defective image. The outcomes of noise and data loss attacks are displayed in Figure 17 and Figure 18, respectively. The deciphered images have a well-visual quality with no lack of visual details inside the area of the cutting portion.

## 7. Conclusions

A novel chaotic flow was proposed in this paper in which its attractor was around some predefined manifolds. The bifurcation diagram of the system has exhibited a period-doubling route to chaos by modifying parameter *a*. Lyapunov exponents of the system were presented to determine the chaotic interval of the parameter. The multistability of the system was revealed by plotting bifurcation diagrams using different initiation methods. The basin of attraction of the attractors was studied. The proposed system was used in a multimedia image cryptosystem. The results were examined using different analyses such as randomness, correlation, plain image sensitivity, key sensitivity, histogram, entropy, and data damage analyses. The results of these tests confirm the reliability of using the presented cryptosystem for various IoT applications from image capture to use it.

## Figures and Tables

**Table 1:** Comparison of time encryption for the presented cryptosystem with other related cryptosystems, as reported in [30,31,32].

Image Cryptosystem	Number of Encrypted Bits Per Second
Proposed	9,713,394
Ref. [30]	8,025,072
Ref. [31]	6,381,110
Ref. [32]	4,224,509

**Table 2:** Outcomes of randomness test.

Test-Name	*p*-Value		Passed
	Key Stream	Cipher-Macaws	
Runs	0.7913189	0.5294244	√
DFT	0.6543473	0.6036850	√
Linear complexity	0.7024604	0.1581112	√
Block-frequency	0.5686530	0.3487918	√
Frequency	0.6559749	0.4300353	√
Universal	0.3457104	0.9949709	√
Serial test 1	0.3920807	0.3989737	√
Serial test 2	0.5967691	0.5770354	√
Overlapping templates	0.7827032	0.4365690	√
No overlapping templates	0.9636127	0.5941496	√
Long runs of ones	0.3969673	0.7129090	√
Approximate entropy	0.2772607	0.1420810	√
Rank	0.0996206	0.5740640	√
Random excursions variant x = 1	0.0963609	0.6213966	√
Random excursions x = 1	0.3570832	0.8722024	√
Cumulative sums (reverse)	0.0620619	0.5399098	√
Cumulative sums (forward)	0.1740884	0.7368549	√

**Table 3:** Correlation coefficient of neighboring pixels for the experimented images.

Image	Direction								
	Hor.			Ver.			Dia.		
	R	G	B	R	G	B	R	G	B
Macaws	0.98685	0.98074	0.98562	0.98874	0.98460	0.98550	0.98024	0.97349	0.97804
Cipher-Macaws	−0.00001	−0.00005	0.00073	0.00026	0.00051	−0.00078	−0.00010	0.00056	−0.00125
Chalet	0.93698	0.92077	0.91862	0.94179	0.93844	0.92290	0.91195	0.90044	0.89372
Cipher-Chalet	0.00083	−0.00002	0.00006	0.00044	−0.00044	0.00114	−0.00017	−0.00018	0.00058
Window	0.95772	0.94235	0.95532	0.97114	0.96285	0.96658	0.93616	0.92107	0.93504
Cipher-Window	0.00087	−0.00088	−0.00066	0.00012	0.00062	0.00144	0.00007	0.00033	−0.00008
Houses	0.92373	0.92224	0.90793	0.88870	0.88994	0.86135	0.82085	0.81976	0.77976
Cipher-Houses	0.00085	−0.00004	−0.00066	0.00059	0.00072	0.00061	−0.00040	0.00128	−0.00075

**Table 4:** Outcomes of NPCR and UNCI.

Image	NPCR	UNCI
Macaws	99.60556%	33.49106%
Chalet	99.61565%	33.45562%
Window	99.60988%	33.47213%
Houses	99.60751%	33.44994%

**Table 5:** $\chi^{2}$ outcomes for the investigated images.

Image	$\chi^{2}$ Value			Outcome
	Red	Green	Blue	
Macaws	303,687.9661	254,324.4518	603,349.4934	Irregular
Chalet	817,766.5013	677,362.4271	616,936.1705	Irregular
Window	515,942.4335	551,843.4661	728,008.0781	Irregular
Houses	285,277.4635	219,680.6289	221,228.6081	Irregular
Cipher-Macaws	244.5221	251.6953	233.6315	Regular
Cipher-Chalet	258.3451	215.8021	243.8919	Regular
Cipher-Window	209.8372	277.1874	254.2682	Regular
Cipher-Houses	226.9335	248.9882	241.1497	Regular

**Table 6:** Local and global information entropies.

Image	Global Entropy		Local Entropy	
	Plain	Cipher	Plain	Cipher
Macaws	7.601941	7.999837	5.396978	7.902671
Chalet	7.136653	7.999834	5.673345	7.903744
Window	7.309858	7.999855	5.441143	7.902341
Houses	7.673795	7.999874	6.552062	7.902232

**Table 7:** Statistical analyses of the cipher all-black and all-white images.

Image	Correlation			$\chi^{2}$ Value	Entropy	
	Hor.	Ver.	Dia.		Global	Local
All-black	−0.0002	−0.0004	0.0001	267.7656	7.99926	7.90340
All-white	0.0006	0.0001	0.0004	226.8008	7.99937	7.90343

## Data Availability

The datasets generated and analysed during the current study are available from the corresponding author upon reasonable request.
